# Supplementary material for: Divide, conquer and reconstruct: How to solve the 3D structure of recalcitrant Micro-Exon Gene (MEG) protein from Schistosoma mansoni
Source: PLoS One. 2023 Aug 3;18(8):e0289444. doi: 10.1371/journal.pone.0289444 (PMC10399815; doi:10.1371/journal.pone.0289444)
Supplement: S1 File — (DOCX) [file pone.0289444.s001.docx]

Supporting information

Divide, conquer and reconstruct: how to solve the 3D structure of recalcitrant Micro-Exon Gene (MEG) protein from *Schistosoma mansoni*

**Stepanka Nedvedova^1,2,3^, Florence Guillière^1^, Adriana Erica Miele^1,4^, François-Xavier Cantrelle^6^, Jan Dvorak^3,5,7^, Olivier Walker^1^ and Maggy Hologne^1,*^**

^1^ Université de Lyon, CNRS, UCB Lyon1, Institut des Sciences Analytiques, UMR5280, 5 rue de la Doua, Villeurbanne 69100, France

^2^ Department of Chemistry, Faculty of Agrobiology, Food and Natural Resources, Czech University of Life Sciences Prague, Kamycka 129, Prague, CZ-16521 Czech Republic

^3^ Department of Zoology and Fisheries, Center of Infectious Animal Diseases, Czech University of Life Sciences, Prague, Kamycka 129, Prague, CZ-16521 Czech Republic

^4^Department of Biochemical Sciences, Sapienza University of Rome, P.le Aldo Moro 5, 00185 Rome, Italy

^5^ Institute of Organic Chemistry and Biochemistry, Czech Academy of Sciences, Prague, Czechia

^6^ Université de Lille, CNRS, UMR8576 – UGSF – Unité de Glycobiologie Structurale et Fonctionnelle, F-59000 Lille

^7^ Faculty of Environmental Sciences, Center of Infectious Animal Diseases, Czech University of Life Sciences in Prague, Kamycka 129, Prague, CZ-16521 Czech Republic

**Expression and purification of MEG 2.1 isoform 1 in bacteria**

*Schistosoma mansoni* MEG 2.1 isoform 1 (Uniprot ID D7PD78) was inserted into the commercial plasmid pET22b(+) between NcoI and XhoI and into pET-SUMO-champion. In both cases a 6xHis tag was present to perform purification by affinity chromatography on Nickel loaded resin. The plasmids were transformed in the following commercial chemically competent expression strains of *Escherichia coli* BL21-Gold (DE3) (Novagen®), One Shot™ BL21(DE3)pLysS (Thermo Fisher Scientific), Rosetta (DE3) (Novagen®), Rosetta(DE3)pLysS (Novagen®). The strain BL21 (DE3) is suitable for T7 containing plasmids as pET series. Since the cells were dying after induction of the expression by IPTG, we first tested the pLysS to limit the toxicity. Since the codons were not optimized for bacterial expression, we used Rosetta competent cells without and with pLysS.

*MEG 2.1 isoform 1 synthetic insert for* ***pET-22b(+)*** *plasmid – with TEV (bold and underlined) and 6xHis-tag (underlined) sequences*

CAG CCG GCG ATG GCC |ATG AAG TTA TCC GGC GCA AAC TGT TTA GTC GTA TTC AGT CTT CTT CAG TTA CTT GTT GCT TTT TCA CAC TGC GAC ATA AAC GAC ATA ACA TGC AAC AAA ACG GTA TGC TGC GCT TCC GAG GAT GGA AAA AAG GGT TCT TTA TGT TGC GAG AAG GAT GGC TGT CCA ATC CCC TCA ACA CCA GAC CTT TTG CTG GGC AAT TAT CAA AGG CAC CAG AGA ATG AAG AAT TAC CTG GAA GAG GTC TGT GAA AAC TTT ATC TAC ACA CCC **GAA AAC CTG TAC TTC CAA|GCG** TTC GAG CAC CAC CAC CAC CAC CAC tga

Primers for amplification:

forward (NcoI) – **CAG CCG GCG ATG GCC** ATG AAG TTA TCC GGC GCA AAC TGT TTA, Tm = 59.3 °C

reverse (XhoI) – **GGT GGT GGT GCT CGA** ACG CTT GGA AGT ACA GGT TTT CAC, Tm = 60.6 °C

bold and underlined – adaptor parts to pET-22b(+) plasmid

*MEG 2.1 isoform 1 synthetic insert for* ***pET SUMO Champion*** *plasmid (bold) – with the preceding SUMO protein sequence*

ATGTCGGACTCAGAAGTCAATCAAGAAGCTAAGCCAGAGGTCAAGCCAGAAGTCAAGCCTGAGACTCACATCAATTTAAAGGTGTCCGATGGATCTTCAGAGATCTTCTTCAAGATCAAAAAGACCACTCCTTTAAGAAGGCTGATGGAAGCGTTCGCTAAAAGACAGGGTAAGGAAATGGACTCCTTAAGATTCTTGTACGACGGTATTAGAATTCAAGCTGATCAGACCCCTGAAGATTTGGACATGGAGGATAACGATATTATTGAGGCTCACAGAGAACAGATTGGTGGT**ATGAAGTTATCCGGCGCAAACTGTTTAGTCGTATTCAGTCTTCTTCAGTTACTTGTTGCTTTTTCACACTGCGACATAAACGACATAACATGCAACAAAACGGTATGCTGCGCTTCCGAGGATGGAAAAAAGGGTTCTTTATGTTGCGAGAAGGATGGCTGTCCAATCCCCTCAACACCAGACCTTTTGCTGGGCAATTATCAAAGGCACCAGAGAATGAAGAATTACCTGGAAGAGGTCTGTGAAAACTTTATCTACACACCCTGA**

forward primer for amplification – ATG AAG TTA TCC GGC GCA AA; Tm = 55 ºC

reverse primer for amplification – TCA GGG TGT GTA GAT AAA GTT; Tm = 50.8 ºC

After transformation into the competent expression strains, the selection was performed on a solid LB medium supplemented with 100 mg/ml of Ampicillin. Single colonies were used for expression in liquid LB medium supplemented with Ampicillin and glucose (final concentration 10 g/L) to keep the expression the most stringent as possible. A range of expression conditions was tested: different expression temperatures (37°C, 30°C, 18°C), different IPTG concentrations (0.1 mM, 0.5 mM, 1 mM), and different starting concentrations for induction (Abs^600^ = 0.6, 0.8, 1.0). Regardless of the number of conditions tested, very poor or no expression was observed. Most of the time the bacterial cells die after induction.

To check for expression whenever the cells survived to 3h or overnight culture, bacteria were harvested by centrifugation at 3000 g for 15 min at room temperature. The pellet was then resuspended in lysis buffer (20 mM Tris/HCl pH 8; 5 mM imidazole; 0.5 M NaCl; 5 mM β-Mercaptoethanol; 10% glycerol; cOmplete™ Protease Inhibitor Cocktail, EDTA-free (Roche)) and the content was sonicated on ice in a metal beaker (amplitude 70, total time 2 min). After sonication, centrifugation was performed at 15 000 g for 30 minutes at 4°C. The supernatant was used to analyze the soluble fraction, and the pellet was further used to analyze the insoluble fraction on SDS-PAGE (Figures S1, S2). The supernatant was then loaded onto a 1 ml or 5 ml (depending on the volume of the culture) Cytiva HisTrap™ FF Column equilibrated with loading buffer at 1 mL/min. After double recirculation of the flow through, gradient elution (5% B, 10% B, 20% B, 50% B, 100% B) and fractionation of 1 mL of sample were performed. The elution buffer contained 20 mM Tris/HCl pH 8; 0.5 M imidazole; 0.5 M NaCl; 10 % glycerol; 5 mM β-Mercaptoethanol. Purification was performed either by gravity flow or by FPLC on AKTA pure system (Cytiva).


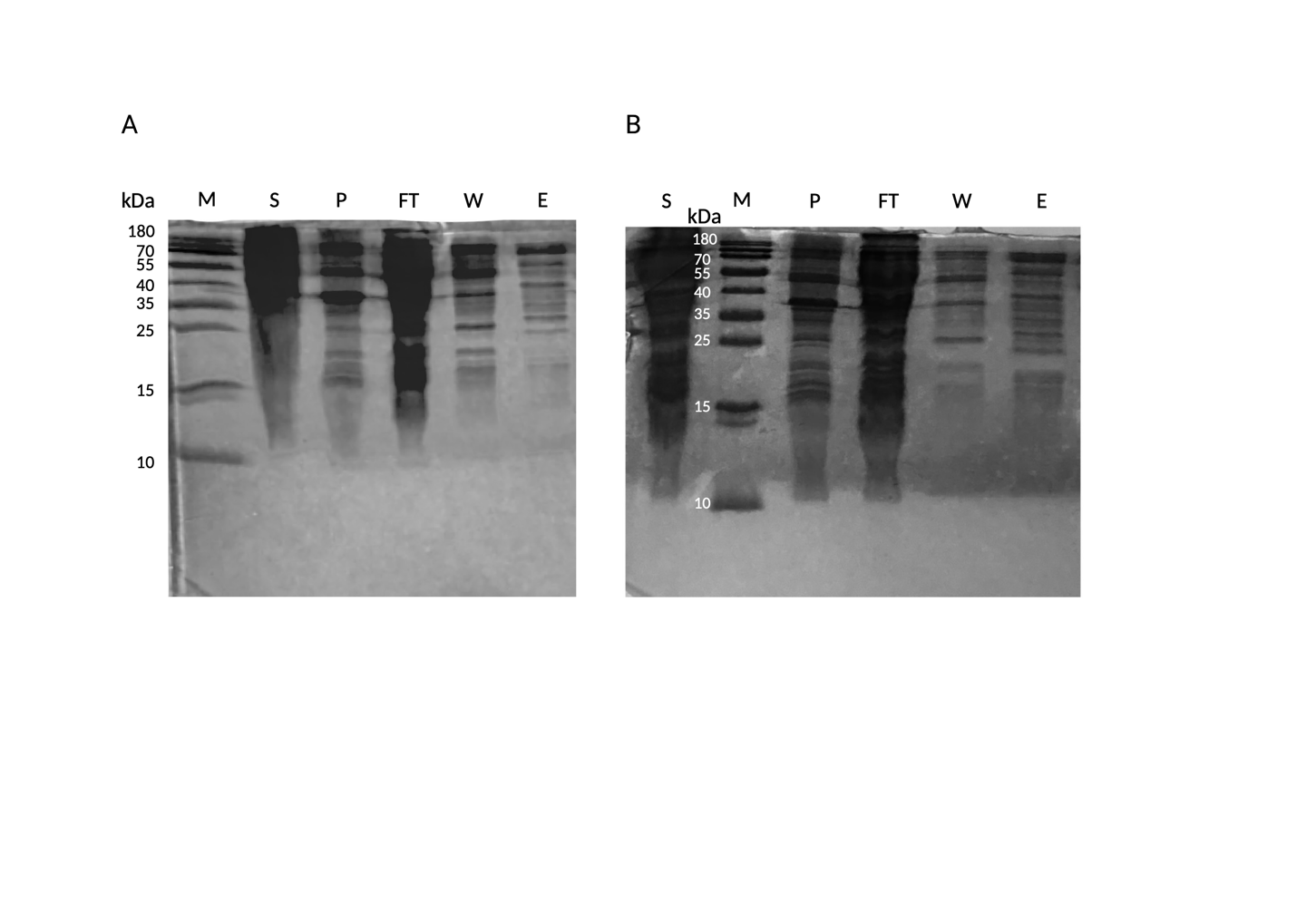


**Figure S1.** SDS-PAGE gel of MEG 2.1 protein – soluble fraction purification with Ni-NTA gravity column; A) in pET-22b(+) plasmid; B) in pET SUMO Champion plasmid

With M = protein molecular weight marker, S = supernatant after cell lysis and centrifugation, P = pellet after centrifugation, FT = flow-through after Ni-NTA loading, W = wash of the column (5-column volume with loading buffer), E = elution with 100% of elution buffer. The expected MEG 2.1 isoform 1 molecular weight is 11.96 kDa for the pET-22b(+) plasmid and 21.04 kDa for the pET SUMO Champion plasmid.


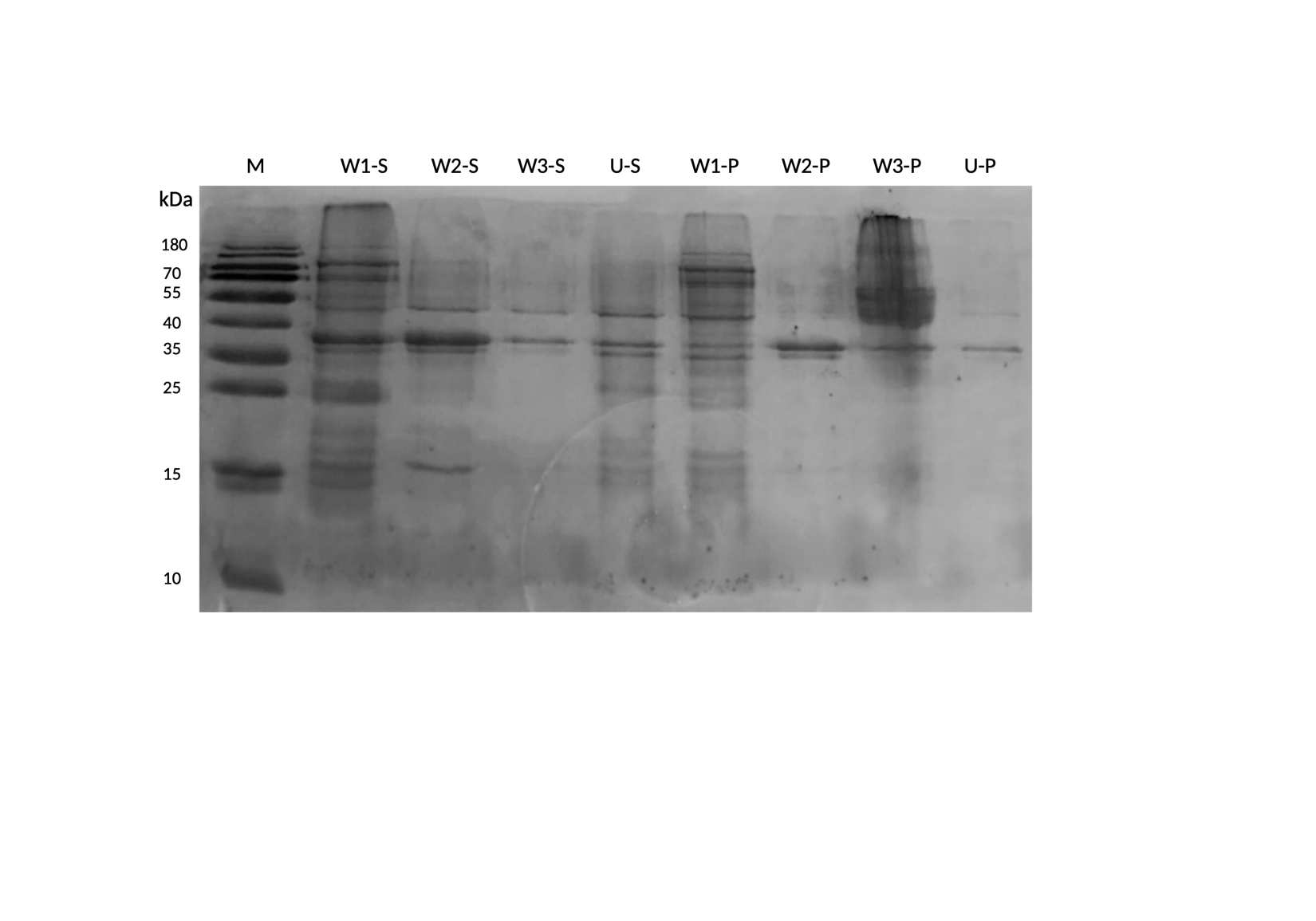


**Figure S2.** SDS-PAGE gel of MEG 2.1 isoform 1 – insoluble fraction purification with Ni-NTA gravity column (W1-S; W2-S; W3-S; U-S – expression from pET SUMO Champion, expected MW= 21.04 kDa), W1-P; W2-P; W3-P; U-P – expression from pET-22b(+), expected Mw = 11.96 kDa).

With M = protein molecular weight marker; W1 = wash and sonication 2 M urea, 20 mM Tris/HCl pH 8, 0.5 M NaCl, 10 mM imidazole, 2% Triton X-100; W2 = wash and sonication in 2 M urea, 20 mM Tris/HCl pH 8, 0.5 M NaCl, 10 mM imidazole; W3 = wash and sonication in 20 mM Tris/HCl pH 8, 0.5 M NaCl, 10 mM imidazole; U = overnight dissolving in 8 M urea.

**Expression and purification of MEG 2.1 isoform 1 in yeast and cell-free system**

The gene for *S. mansoni* MEG 2.1 isoform 1 was optimized for expression in the methanotrophic yeast *Komagatella pastoris*. We inserted the gene in the pPICZαB plasmid in the PstI site before the 6xHis tag.

*MEG 2.1 isoform 1 synthetic insert for pPICZα B plasmid*

GAGAAAGAGAGGCTGAAGCTGCA AAG TTA TCC GGC GCA AAC TGT TTA GTC GTA TTC AGT CTT CTT CAG TTA CTT GTT GCT TTT TCA CAC TGC GAC ATA AAC GAC ATA ACA TGC AAC AAA ACG GTA TGC TGC GCT TCC GAG GAT GGA AAA AAG GGT TCT TTA TGT TGC GAG AAG GAT GGC TGT CCA ATC CCC TCA ACA CCA GAC CTT TTG CTG GGC AAT TAT CAA AGG CAC CAG AGA ATG AAG AAT TAC CTG GAA GAG GTC TGT GAA AAC TTT ATC TAC ACA CCC ggt CATCATCATCATCATCATTAAGGAATTCACGTGGCCCAG

forward primer for amplification – GAGAAAGAGAGGCTGAAGCTGCA; Tm = 59 °C

reverse primer for amplification – CTG GGC CAC GTG AAT TCC TTA ATG; Tm = 58 °C

In an attempt to produce MEG 2.1 isoform 1 and MEG 3.2 in a cell-free system, the genes with and without the putative N-terminal signal peptides were inserted into the plant-derived plasmid pIVEX2.4d between NotI and BamHI.

*MEG 2.1 isoform 1 with signal peptide (pIVEX2.4d-TEV-PS-MEG2.1)*

gcggccgcgagaatctttattttcagggcATGAAACTATCGGGAGCAAACTGTTTGGTAGTCTTCAGCCTACTACAACTTCTTGTGGCATTTTCACACTGTGATATTAATGACATAACATGCAACAAGACAGTTTGTTGCGCATCAGAAGACGGTAAAAAAGGTTCCCTATGTTGTGAGAAAGATGGTTGTCCAATTCCAAGCACTCCAGATCTTTTGCTTGGAAATTACCAGCGCCATCAACGAATGAAAAATTATTTAGAGGAAGTGTGCGAAAATTTCATATACACGCCATAATAAggatcc

*MEG 2.1 isoform 1 without signal peptide (pIVEX2.4d-TEV-MEG2.1)*

gcggccgcgagaatctttattttcagggcGATATTAATGACATAACATGCAACAAGACAGTTTGTTGCGCATCAGAAGACGGTAAAAAAGGTTCCCTATGTTGTGAGAAAGATGGTTGTCCAATTCCAAGCACTCCAGATCTTTTGCTTGGAAATTACCAGCGCCATCAACGAATGAAAAATTATTTAGAGGAAGTGTGCGAAAATTTCATATACACGCCATAATAAggatcc

*MEG 3.2 isoform 1 with signal peptide (pIVEX2.4d-TEV-PS-MEG3.2)*

gcggccgcgagaatctttattttcagggcATGCTGTTCGTTGCATTGATTCTGATCATCTCTCTCCACTCATTCGACTGTGTATTCACAGCTCGTGAAACTCAACAAGAATGTGTACGACATTGTGGTGGACACAATGAATATGTGACTCGATACTGTGGTGGTCTGTGTTCTGGCAGCACAGGACCACAAACATTCTATTGTTATCTCGGATGCAGTCATAACGCCAGTAACCAAAACGATTTCGACAAATGTTTACCAAAGTGTAATGGTAGTCCCCAGCTTACTGAGTCATCGTGTCAGAATGACTGTGGTCGTGTTACCACACACCCTGAATTGTGTGGTATCGTTTGTGGTGGAAATGTTGGAGACTCATTTCCACTGTGTTTGTATAACTGCGATCAGGGAAATGGTTCGGGAAACTTTGACGAATGTAAAACAAAGTGCTACGAAATGGCGGGACGGTGATAAggatcc

*MEG 3.2 isoform 1 without signal peptide (pIVEX2.4d-TEV-MEG3.2)*

gcggccgcgagaatctttattttcagggcGCTCGTGAAACTCAACAAGAATGTGTACGACATTGTGGTGGACACAATGAATATGTGACTCGATACTGTGGTGGTCTGTGTTCTGGCAGCACAGGACCACAAACATTCTATTGTTATCTCGGATGCAGTCATAACGCCAGTAACCAAAACGATTTCGACAAATGTTTACCAAAGTGTAATGGTAGTCCCCAGCTTACTGAGTCATCGTGTCAGAATGACTGTGGTCGTGTTACCACACACCCTGAATTGTGTGGTATCGTTTGTGGTGGAAATGTTGGAGACTCATTTCCACTGTGTTTGTATAACTGCGATCAGGGAAATGGTTCGGGAAACTTTGACGAATGTAAAACAAAGTGCTACGAAATGGCGGGACGGTGATAAggatcc

All the plasmids were transformed into competent *E. coli* cells for midiprep production prior to expression in a cell-free system (Figure S3). LB agar medium supplemented with ampicillin was used for the selection of transformants. A 6xHis tag was present at the N-terminus of each construct. Even in this system the expression was undetectable by western blot (Figure S4).


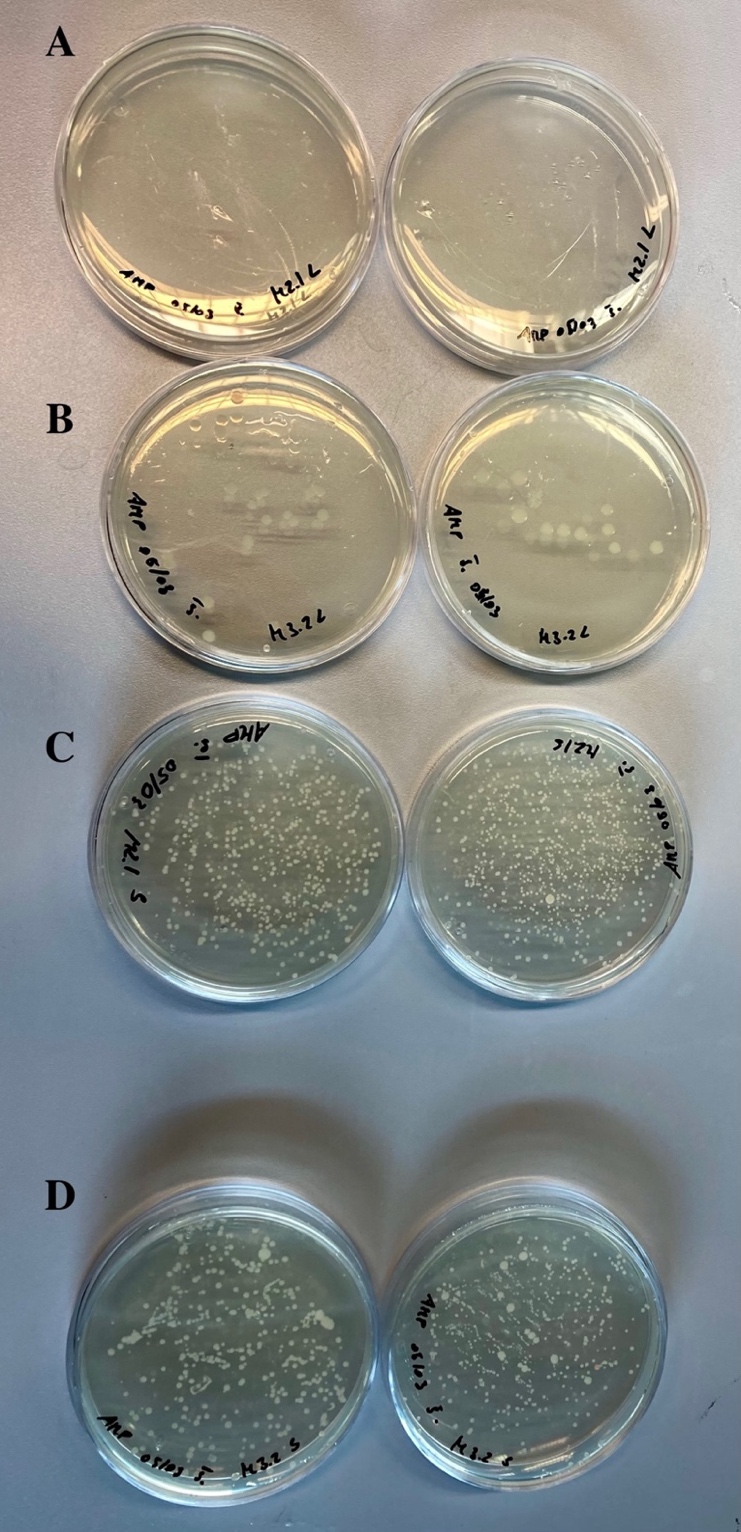


**Figure S3.** *E. Coli* BL21(DE3) transformed with cell-free expression pIVEX2.4d plasmids with MEG 2.1 isoform 1 and MEG 3.2 isoform 1 inserts (design detail above). A) construct pIVEX2.4d-TEV-PS-MEG2.1; B) construct pIVEX2.4d-TEV-MEG2.1; C) construct pIVEX2.4d-TEV-PS-MEG3.2; D) construct pIVEX2.4d-TEV-MEG3.2.


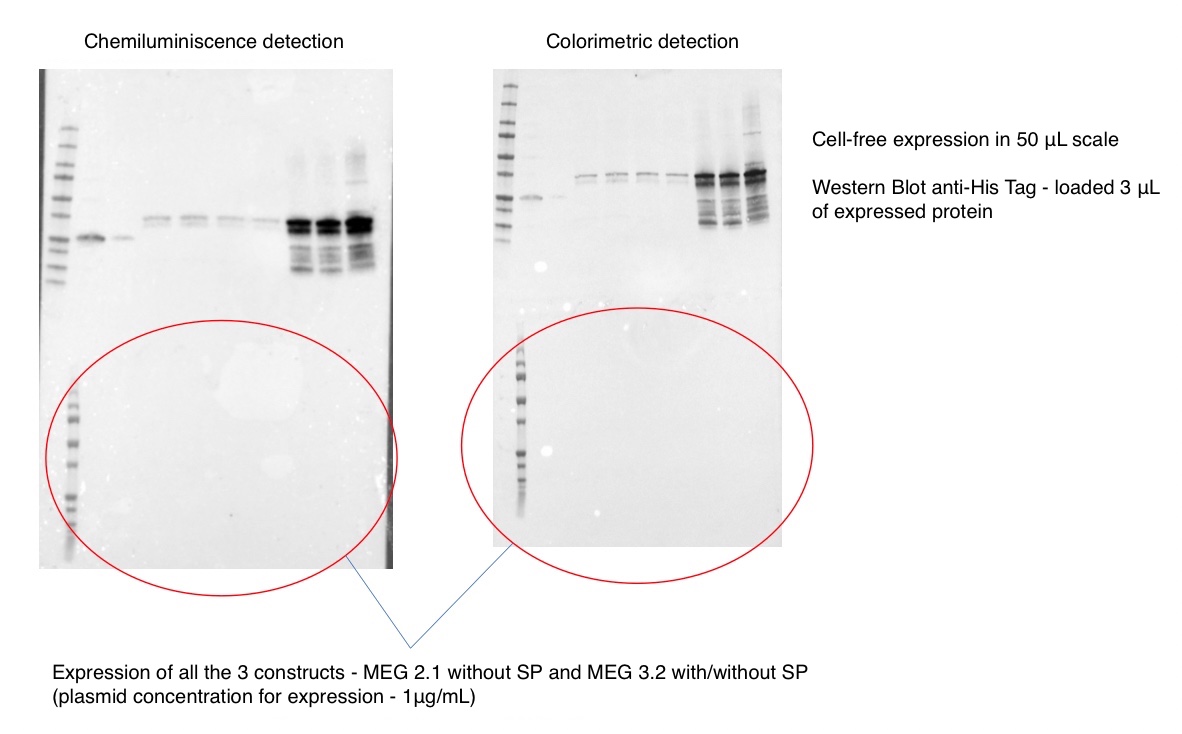


**Figure S4.** Western Blot gels after cell-free expression of three constructs - MEG 2.1 without signal peptide and MEG 3.2 (with and without signal peptide).

**3D structural determination of synthetic peptides derived from MEG 2.1 isoforms by multi-dimensional NMR with natural isotopic abundance.**


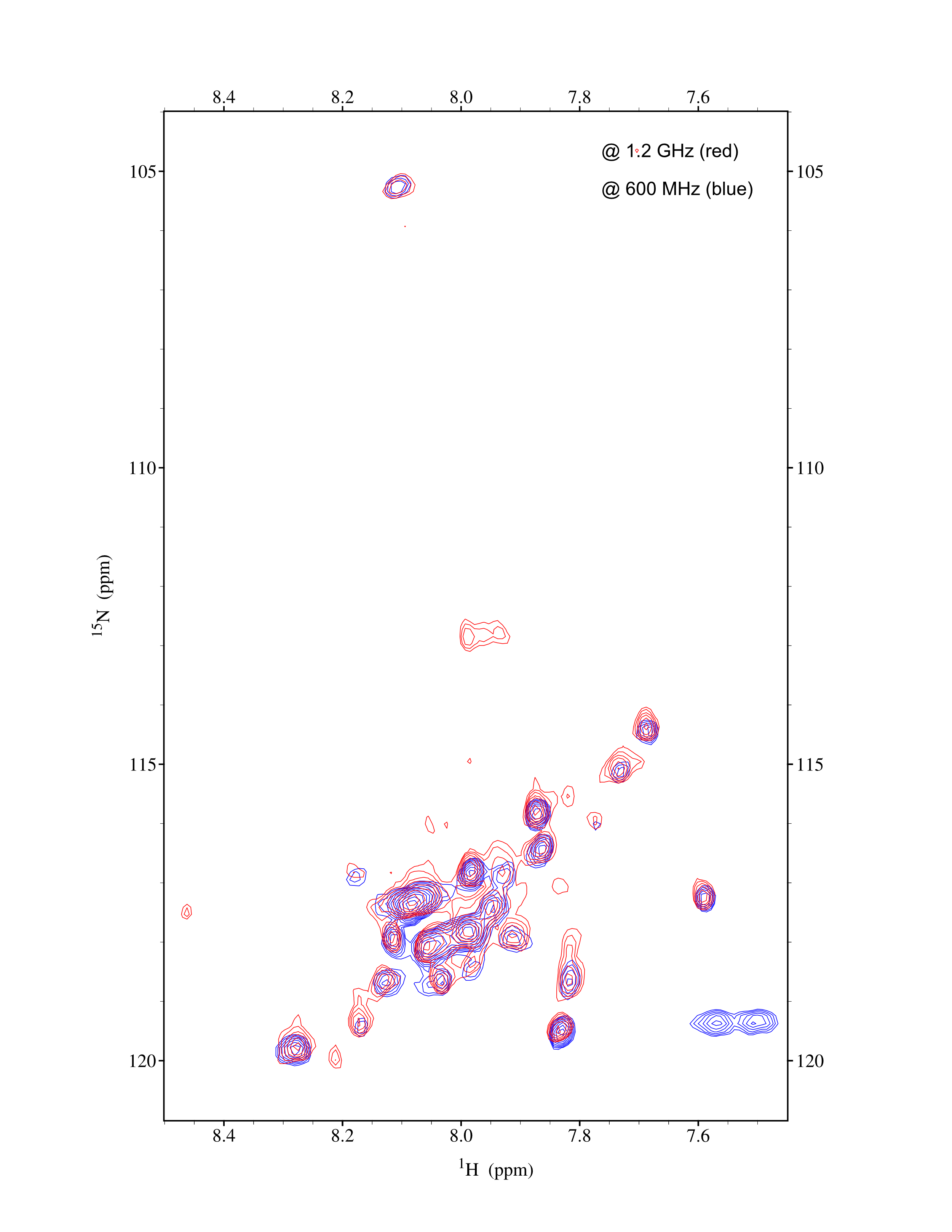


**Figure S5.** Overlay of ^1^H-^15^N HSQC spectra recorded at 27°C and at a ^1^H frequency of 1.2 GHz (red) and 600 MHz (blue) for the isoform 1 at 2 mM dissolved in DMSO-d_6_. The two blue resonances at 119.5 and 7.45 ppm can be assigned to lysine or arginine side chain chemical shifts.


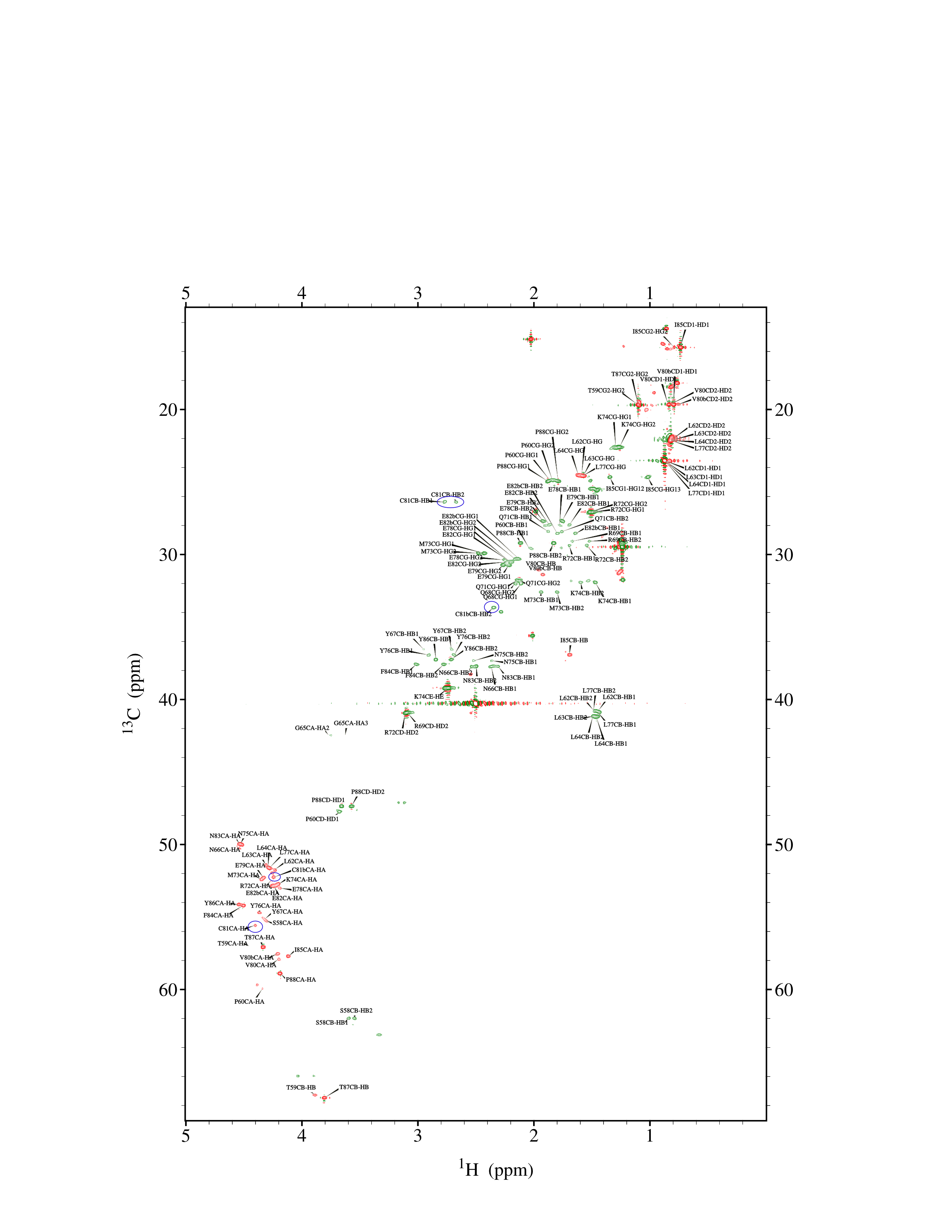


**Figure S6.** 2D ^1^H-^13^C HSQC assignments of MEG 2.1 isoform 1 (25-88) peptide (2mM). The experiment was recorded at 27°C with a Bruker Neo spectrometer operating at a ^1^H frequency of 1.2 GHz and equipped with a triple HCN cryoprobe. The resonances of Cα and Cβ for C81 and C81b are highlighted with a blue circle.

| **Validation of the reconstructed structures (best 10 models)**   1. Isoform 1a   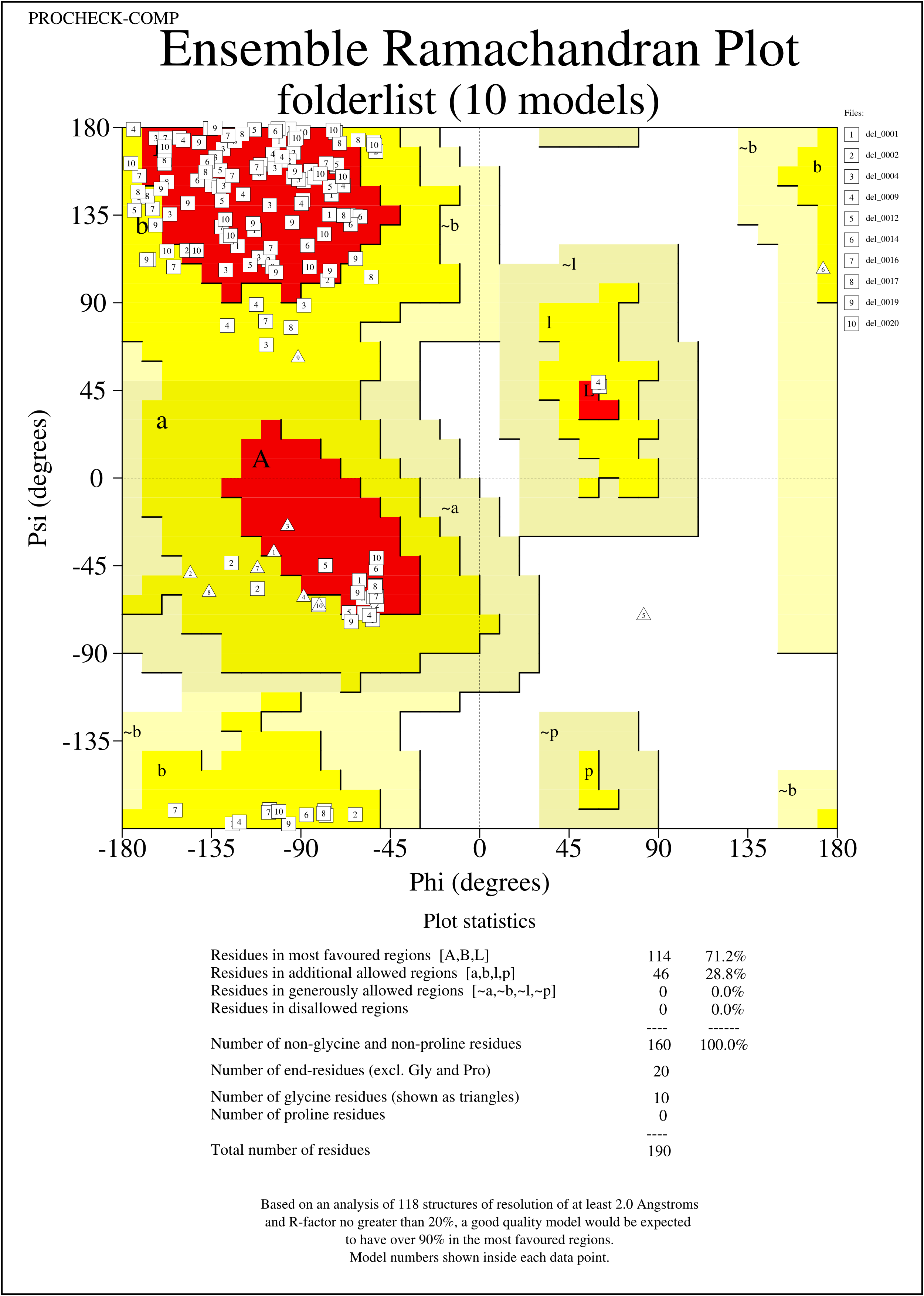 |
| --- |
| 1. Isoform 1b   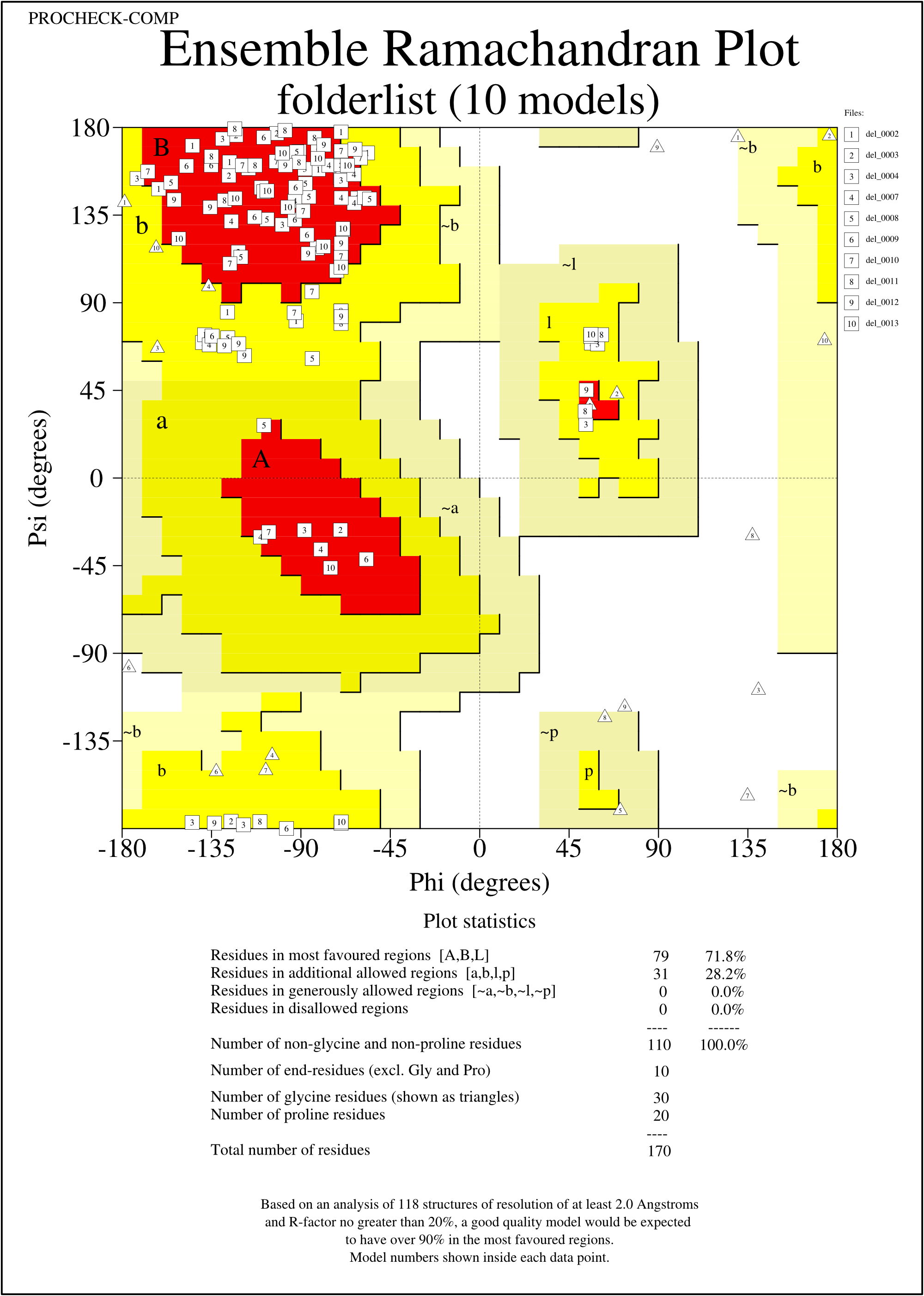 |
| 1. Isoform 1c   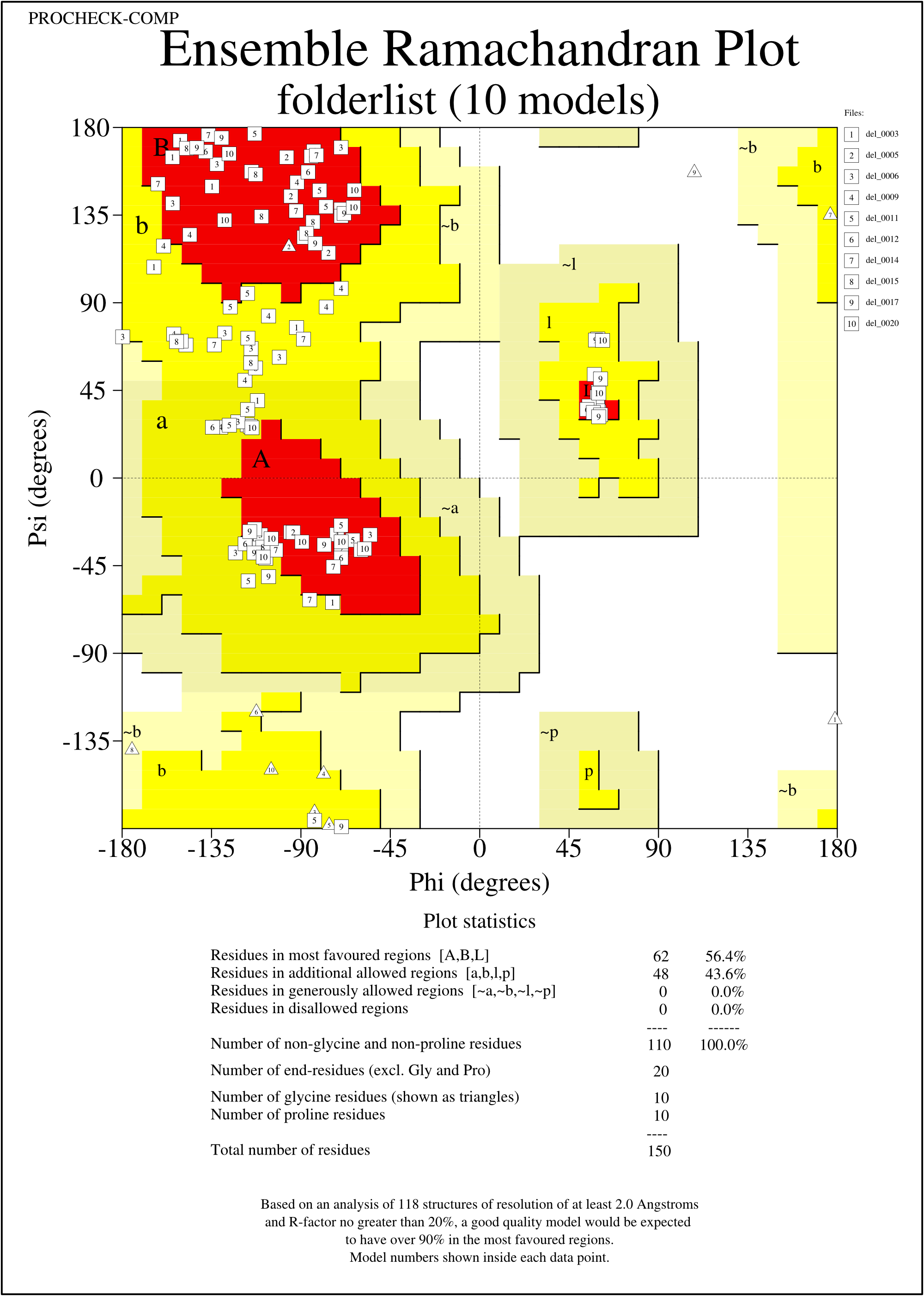 |
| 1. Isoform 1d   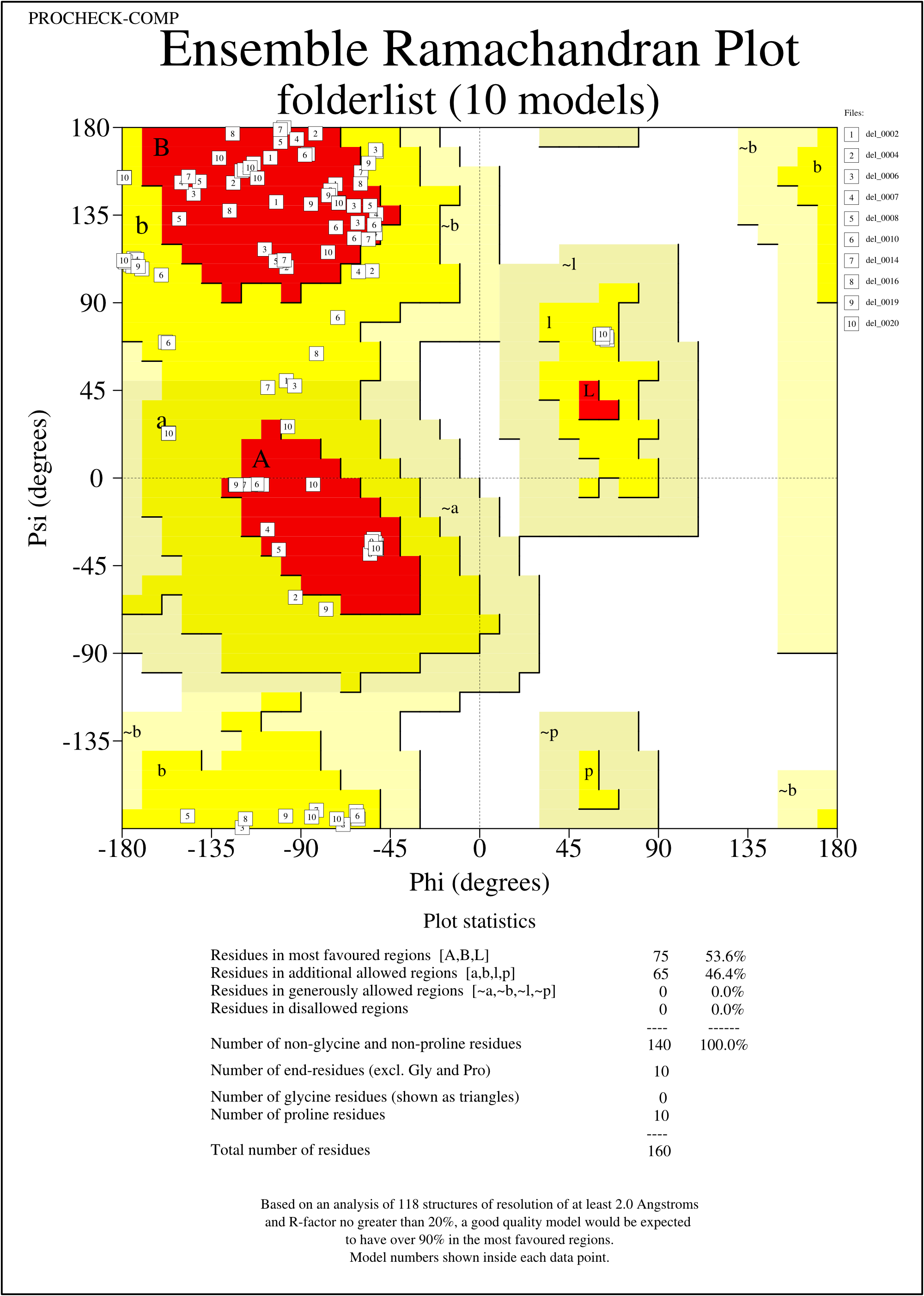 |
| 1. Isoform 2a   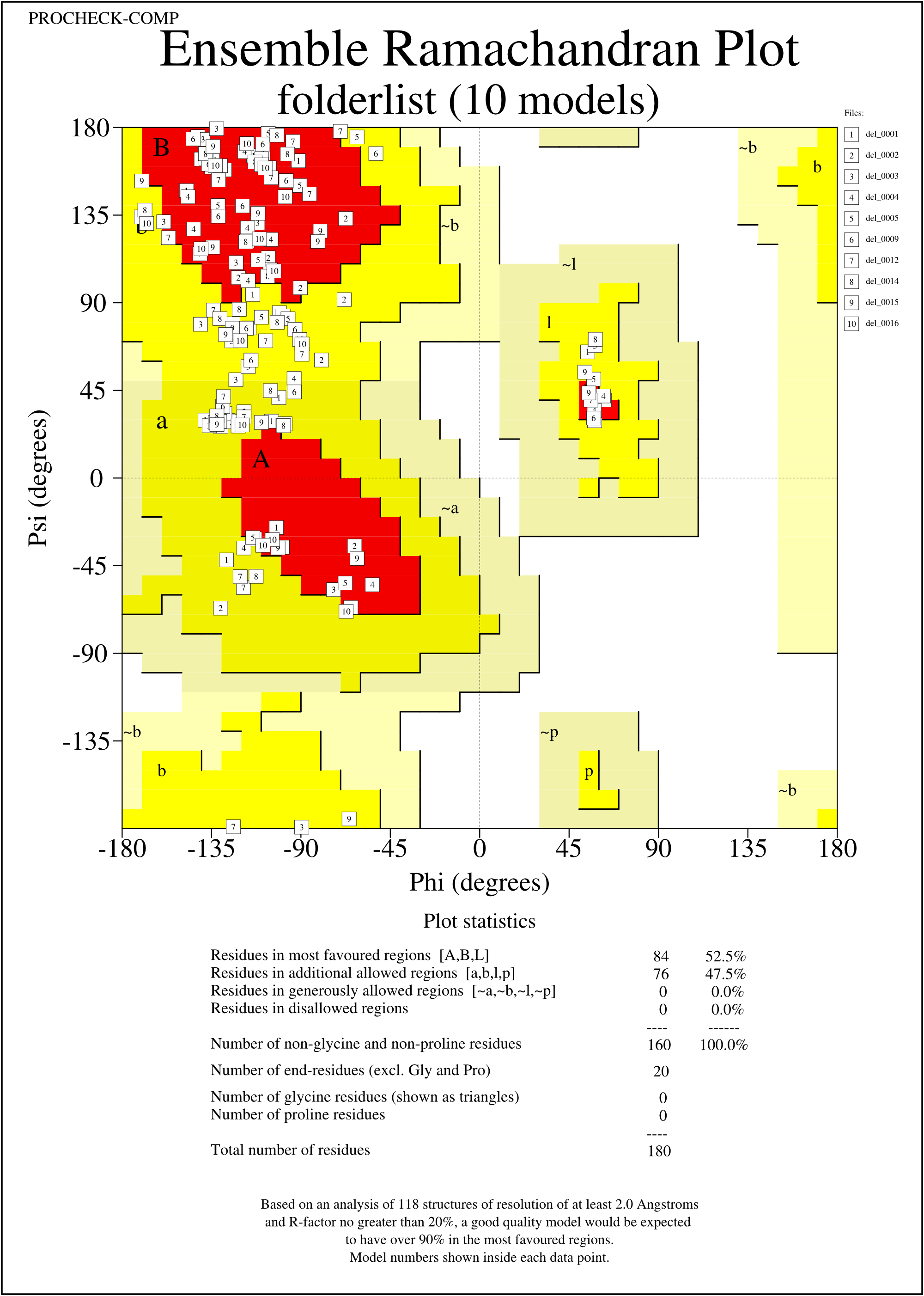 |
| 1. Isoform 2b   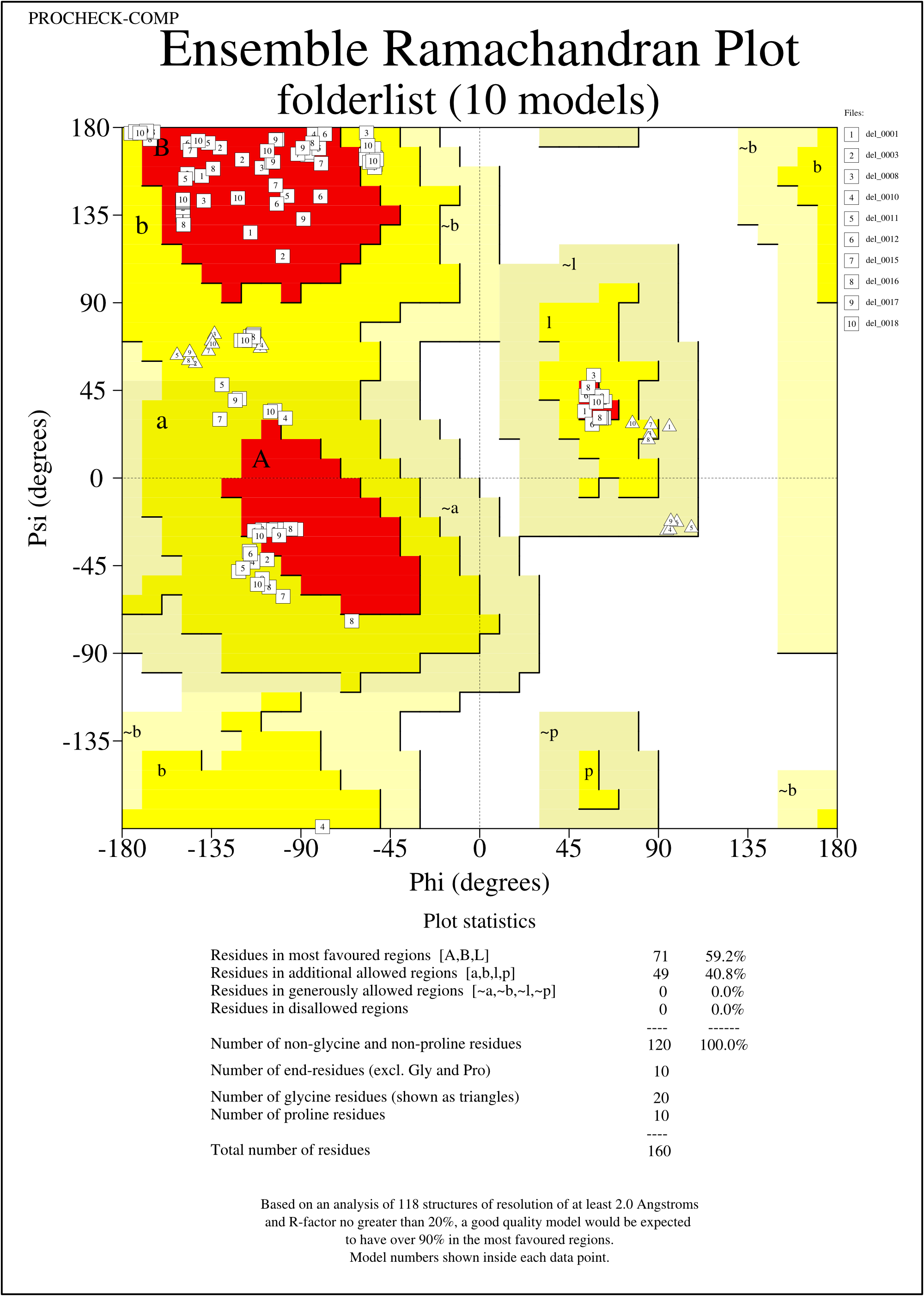 |

**Figure S7.** Ramachandran plots for A) MEG 2.1 iso1a, B) MEG 2.1 iso1b, C) MEG 2.1 iso 1c, D) MEG 2.1 iso1d, E) MEG 2.1 iso 2a and F) MEG 2.1 iso 2b. The repartition of the angles in function of the favored, allowed or disallowed regions is indicated under each plot.


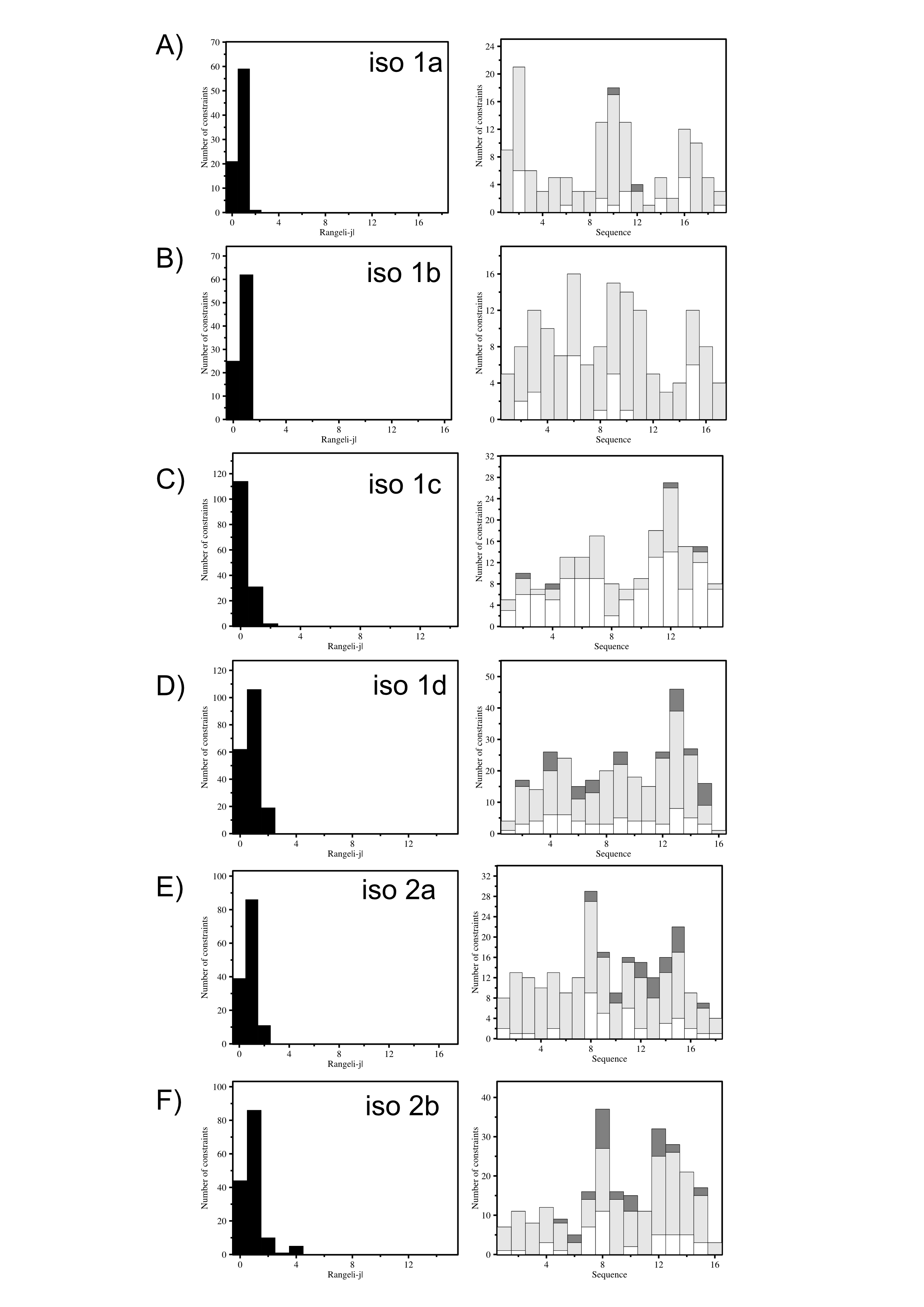


**Figure S8.** Number of NMR constraints distributed according to the distance range (left panel) and to the residue number in the sequence (right panel) for A) MEG 2.1 iso 1a, B) MEG 2.1 iso 1b, C) MEG 2.1 iso 1c, D) MEG 2.1 iso 1d, E) MEG 2.1 iso 2a and F) MEG 2.1 iso 2b. In the right panel, short distances are displayed in white, medium distances in light grey and long distances in dark grey.


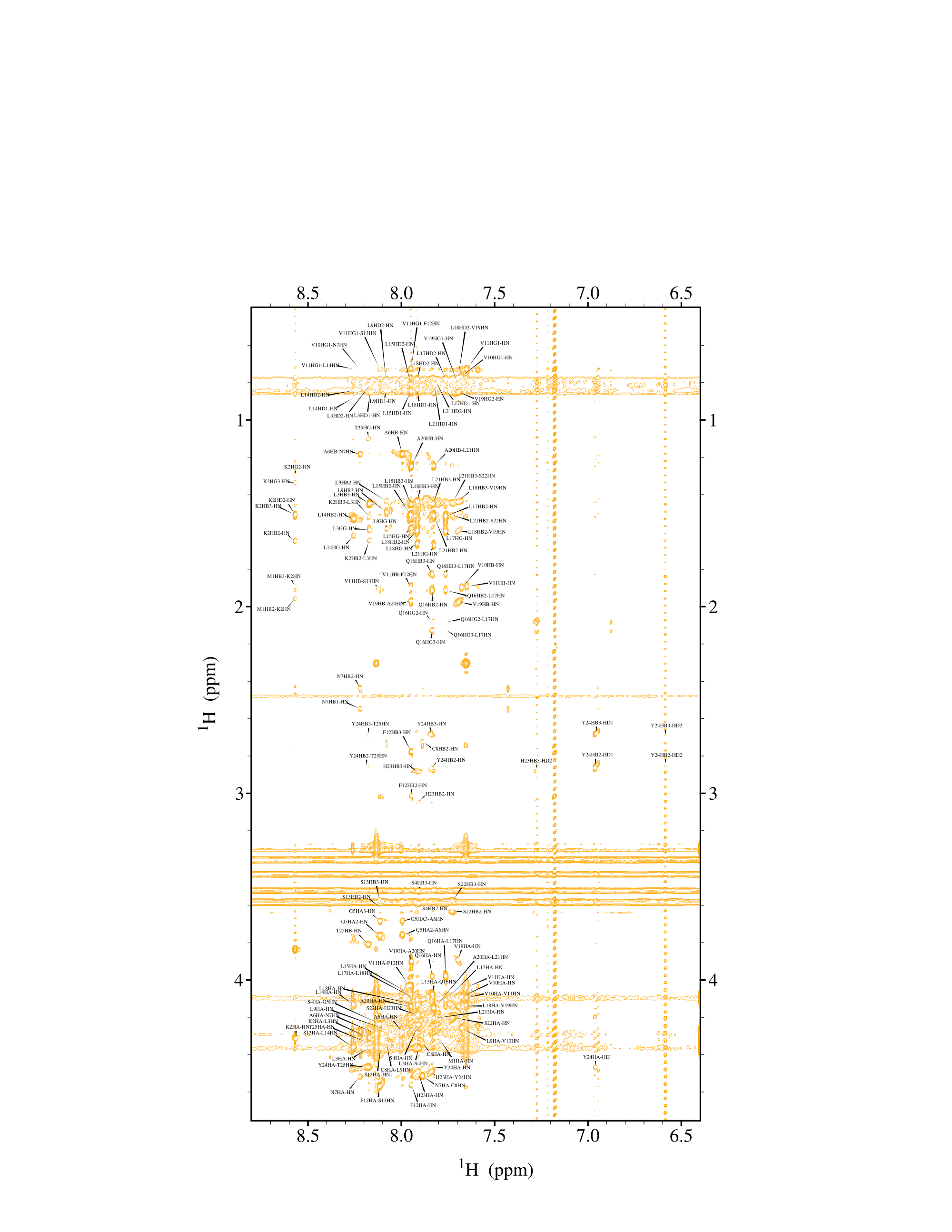


**Figure S9.** H_N_/H_aliphatic_ region of the ^1^H-^1^H NOESY spectrum of isoform 3 (2 mM) recorded at at 27°C with a Bruker Neo spectrometer operating at a ^1^H frequency of 1.2 GHz and equipped with a triple HCN cryoprobe.


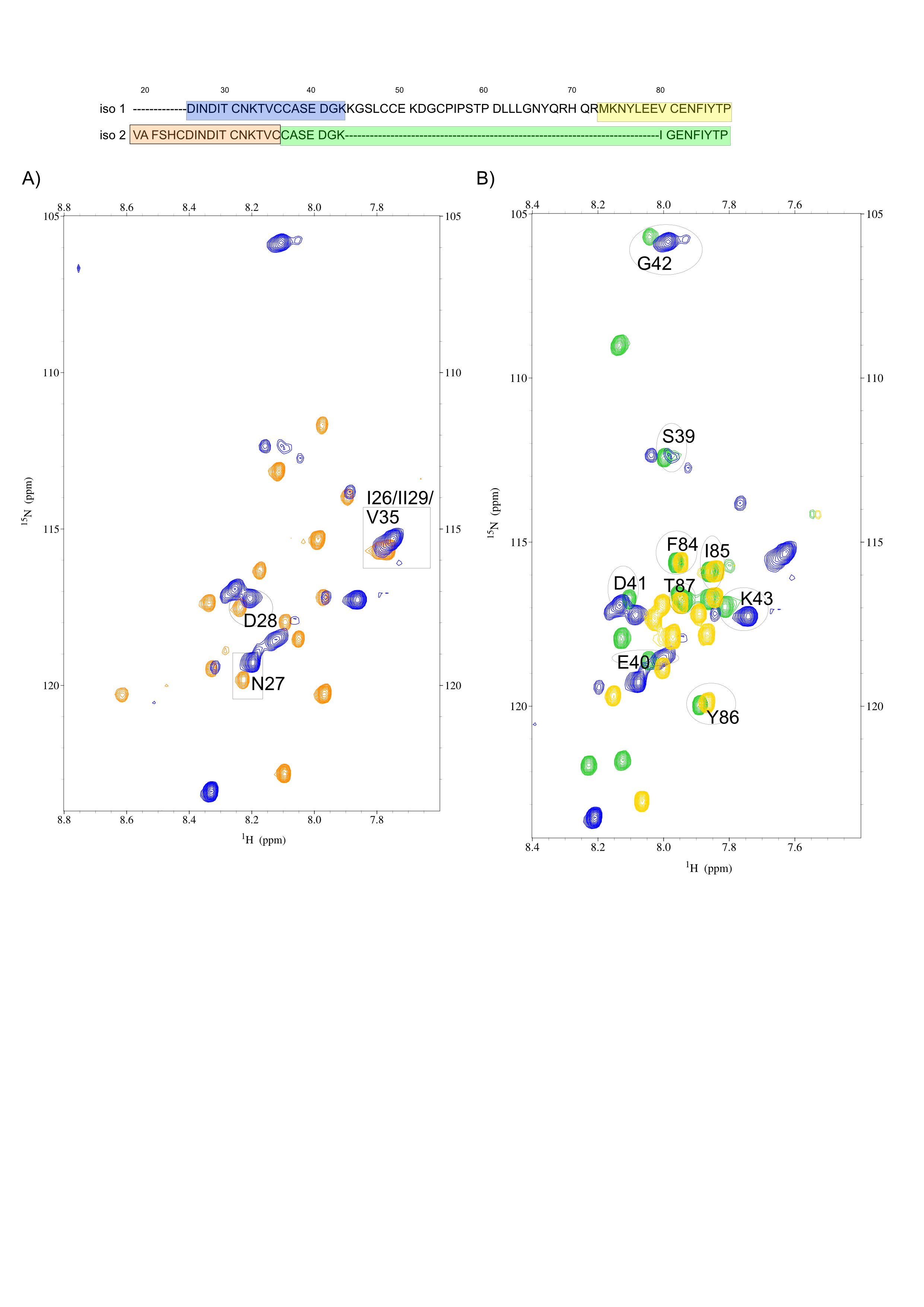


**Figure S10.** Overlay of ^1^H-^15^N HSQC spectra of A) iso 1a (blue) and iso 2a (orange), B) iso 1a (blue), iso 1d (yellow) and iso 2b (green). The spectra were recorded at 27°C with a Varian spectrometer operating at a ^1^H frequency of 600 MHz.

| 1. Isoform 1 (25-88)      1. Isoform 1a     #res2 corresponds to the numbering of the full-length isoform 1.   1. Isoform 1b     #res2 corresponds to the numbering of the full-length isoform 1.   1. Isoform 1c     #res2 corresponds to the numbering of the full-length isoform 1.   1. Isoform 1d     #res2 corresponds to the numbering of the full-length isoform 1.   1. Isoform 3      1. Isoform 2a    |
| --- |
| #res2 corresponds to the numbering of the full-length isoform 2.   1. Isoform 2b    |

#res2 corresponds to the numbering of the full-length isoform 2.

**Table S1.** ^1^H, ^13^C and ^15^N chemical shifts of A) MEG 2.1 isoform 1 (25-88), B) MEG 2.1 isoform 1a, C) MEG 2.1 isoform 1b, D) MEG 2.1 isoform 1c, D) MEG 2.1 isoform 1d, E) MEG 2.1 isoform 3, F) MEG 2.1 isoform 2a and G) MEG 2.1 isoform 2b dissolved in DMSO-d_6_ measured at 27 °C. For each table, the numbering refers to the synthetic peptide order (#res) and the numbering of the full-length isoform is given in #res2.
