# Supplementary material for: Divide, conquer and reconstruct: How to solve the 3D structure of recalcitrant Micro-Exon Gene (MEG) protein from Schistosoma mansoni
Source: PLoS One. 2023 Aug 3;18(8):e0289444. doi: 10.1371/journal.pone.0289444 (PMC10399815; doi:10.1371/journal.pone.0289444)
Supplement: Attachment — Submitted filename: answer-reviewer.docx [file pone.0289444.s002.docx]

Lyon, July 18^th^ 2023

Dr. Barnwal

Academic Editor

Plos One

*Manuscript number:* PONE-D-23-18239

Dear Dr. Barnwal,

Enclosed is our revised manuscript, entitled, “Divide, conquer and reconstruct: how to solve the 3D structure of recalcitrant Micro-Exon Gene (MEG) protein from *Schistosoma mansoni*”. We are pleased with the responses of reviewer and would like to thank him/her for the useful comments that helped us to improve the clarity of our manuscript. Below is a detailed, point by point account of how we addressed the reviewers’ comments (the reviewers’ comments are in bold). Any change in the main text is now highlighted in blue.

Reviewer #1

1. **In the abstract, the authors mentioned that all isoforms of MEG 2.1 were analyzed using both biophysical and computational techniques. However, upon reviewing the manuscript, it is apparent that only isoform 1 was analyzed computationally. Therefore, the authors are advised to revise the sentences accordingly to accurately reflect the scope of their computational analysis.**

Following the reviewers’ comment, we modified the sentence in the abstract: “We demonstrated that the combination of biophysical techniques, like circular dichroism and nuclear magnetic resonance at natural abundance, with *in silico* molecular dynamics simulation for isoform 1 only, was the key to solve the structure of MEG 2.1.”

2. **The manuscript lacks some important figures, such as the root mean square deviation (RMSD), root mean square fluctuation (RMSF), and radius of gyration (Rg) graphs of the molecular dynamics (MD) trajectory. These figures are crucial for assessing the stability of the protein structure, understanding the extent of residue fluctuations, and evaluating the structural compactness of MEG 2.1. As the manuscript focuses on the structure determination of MEG 2.1 isoforms, it is essential to discuss these results and include the corresponding figures.**

We would like to thank the reviewer to bring to our attention these missing information. Therefore, we added a new figure (Figure 7) in the main text showing the RMSD, RMSF and Rg derived from the MD trajectory. A paragraph was also added in the results part to discuss the data assessing the stability of the protein structure as well as a modified sentence in the discussion part (“Moreover, the 4 µs MD simulation on MEG 2.1 isoform 1 (25-88) shows a transition with the formation of a stable α-helix at the N-terminus and of turns/small helices at the C-terminus (Figures 6, 7)”. A sentence was also added in the Materials and Methods part concerning Rg, RMSD and RMSF calculation.

3. **The authors should carefully review the citation of figures within the text, particularly in the MD results and discussion section. It has been observed that some citations do not match the referenced figures, causing confusion. Therefore, the authors are advised to ensure consistency and accuracy in the citation of figures throughout the manuscript.**

Following the recommendation of the reviewer, the consistency and accuracy in the citation of the figures were checked carefully before resubmission.

4. **The article contains grammatical and typographical errors that require scientific editing. It is recommended that the authors carefully review the manuscript to correct these errors and enhance the overall language quality.**

To avoid grammatical and typographical errors, the manuscript was reread carefully, and we tried to do our best to polish our article.

Thus, we believe that we have addressed all reviewers’ concerns.

Sincerely,

Dr. Maggy Hologne

Equipe Biophysics of Complex Systems

Institut des Sciences Analytiques (ISA)

UMR 5280 CNRS - Université Claude Bernard Lyon 1 (UCBL)

5 rue de la Doua

69100 Villeurbanne

France

Phone : +33.4.37.42.35.45

e-mail : maggy.hologne@univ-lyon1.fr
